# Performance Comparison of Classical Methods and Neural Networks for Colour Correction

**DOI:** 10.3390/jimaging9100214

**Published:** 2023-10-07

**Authors:** Abdullah Kucuk, Graham D. Finlayson, Rafal Mantiuk, Maliha Ashraf

**Affiliations:** 1 School of Computing Sciences, University of East Anglia, Norwich NR4 7TJ, UK; g.finlayson@uea.ac.uk; 2 Department of Computer Science and Technology, University of Cambridge, Cambridge CB3 0FD, UK; rafal.mantiuk@cl.cam.ac.uk (R.M.); ma905@cam.ac.uk (M.A.)

**Keywords:** colour correction, regression, polynomial, optimisation, exposure invariance, neural network

## Abstract

Colour correction is the process of converting RAW RGB pixel values of digital cameras to a standard colour space such as CIE XYZ. A range of regression methods including linear, polynomial and root-polynomial least-squares have been deployed. However, in recent years, various neural network (NN) models have also started to appear in the literature as an alternative to classical methods. In the first part of this paper, a leading neural network approach is compared and contrasted with regression methods. We find that, although the neural network model supports improved colour correction compared with simple least-squares regression, it performs less well than the more advanced root-polynomial regression. Moreover, the relative improvement afforded by NNs, compared to linear least-squares, is diminished when the regression methods are adapted to minimise a perceptual colour error. Problematically, unlike linear and root-polynomial regressions, the NN approach is tied to a fixed exposure (and when exposure changes, the afforded colour correction can be quite poor). We explore two solutions that make NNs more exposure-invariant. First, we use data augmentation to train the NN for a range of typical exposures and second, we propose a new NN architecture which, by construction, is exposure-invariant. Finally, we look into how the performance of these algorithms is influenced when models are trained and tested on different datasets. As expected, the performance of all methods drops when tested with completely different datasets. However, we noticed that the regression methods still outperform the NNs in terms of colour correction, even though the relative performance of the regression methods does change based on the train and test datasets.

## 1. Introduction

**Figure 1:**
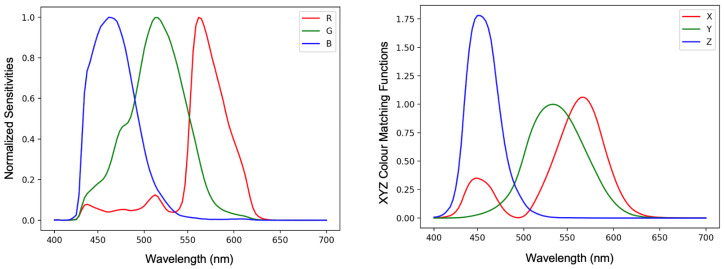
Normalised sensitivity functions of Nikon D5100 camera (**left**) and the CIE XYZ standard observer colour matching functions (**right**). The XYZ matching curves are relative to ‘Y’ (green curve) which has a maximum response of 1.

Colour correction algorithms usually convert camera-specific RGB values into camera-independent colour spaces such as sRGB [1] or CIE XYZ [2]. In Figure 1, we plot spectral sensitivity functions of the Nikon D5100 camera and CIE XYZ colour matching functions. If there existed a linear transform which took the Nikon (or any other camera) sensitivity curves so that they were equal to the XYZ matching function then the same linear transform would perfectly correct the camera’s RGB responses to the corresponding XYZ tristimuli. However, there are no commercial photographic cameras that meet this linear transform condition and so camera RGBs can only be approximately converted to XYZs.

**Figure 2:**
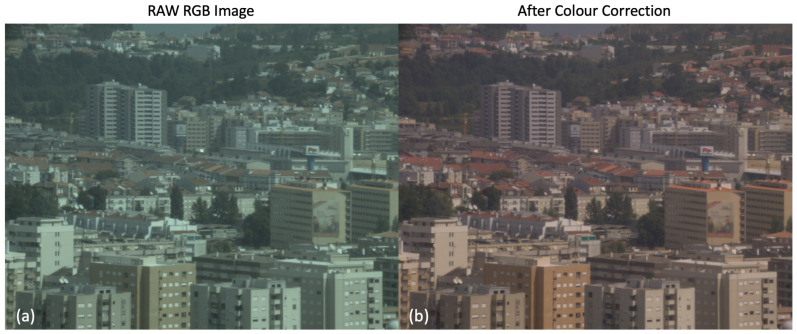
An illustration of colour correction. The images are generated from David Foster’s hyperspectral reflectance dataset [3] with Nikon D5100 camera responses and D65 illumination. The left one (**a**) demonstrates the RAW RGB image; the right one (**b**) represents the colour-corrected sRGB image.

An illustration of the colour correction problem is shown in Figure 2. Here, RAW RGB Nikon D5100 camera responses are converted by linear colour correction to the sRGB [1] colour space. The image shown is drawn from the Foster et al. hyperspectral image set [3] with the RGB and sRGB images calculated by numerical integration. Both images have the sRGB non-linearity applied.

The most common approach to colour correction maps RGB data to corresponding XYZs using a 3 × 3 matrix (found by regression) such that:

(1) $$\begin{array}{c} M\rho \approx x \end{array}$$

where ρ and x represent the RAW RGB camera response vector and XYZ tristimulus, respectively. Polynomial [4] and root-polynomial [5] approaches can also be used for colour correction. In each case, the RGB values are expanded according to the order of the polynomial (normal or root) and a higher order regression is used to determine the regression transform. As an example, the second-order root-polynomial expansion maps ${\left[R\;G\;B\right]}^{T}$ to ${\left[R\;G\;B\;\sqrt{RG}\;\sqrt{RB}\;\sqrt{GB}\;\right]}^{T}$ (${}^{T}$ denotes transpose) and the correction matrix *M* is 3×6.

In addition to regression methods, neural networks have been used for colour correction. In the literature, there are several shallow network approaches [6,7,8], and more recently, convolutional neural networks have been proposed [9,10,11]. However, the recent literature focuses on the problem of correcting the colours captured underwater where the correction problem is different to the one we address here (e.g., they deal with the attenuation of colour due to the distance between the subject and the camera).

Recently, MacDonald and Mayer [12] designed a Neural Net (**NN**) with a fully connected Multilayer Perceptron (MLP) structure for colour correction and demonstrated that the network delivered colour correction that was better than the linear approach (Equation (1)). In the first part of this paper, we investigate the performance of **NN** algorithm versus regression methods more generally. Broadly, we confirm the finding that the **NN** approach is significantly better than linear regression but that the polynomial [4] and root-polynomial [5] regressions actually deliver significantly better colour correction than the **NN** [13].

As well as delivering poorer performance than the best regression methods, the **NN** approach was also found not to be exposure invariant [14]. That is, a network trained to map RGBs to XYZs for a given exposure level delivered relatively poor colour correction when the exposure level changed. The polynomial colour correction algorithm [15] suffers from the same exposure problem; polynomial regression works very well for a fixed exposure but less well when exposure changes [5]. Indeed, the existence of this problem led to the development of the *root* polynomial correction algorithm (which, by construction, is exposure invariant) [5].

**Figure 3:**
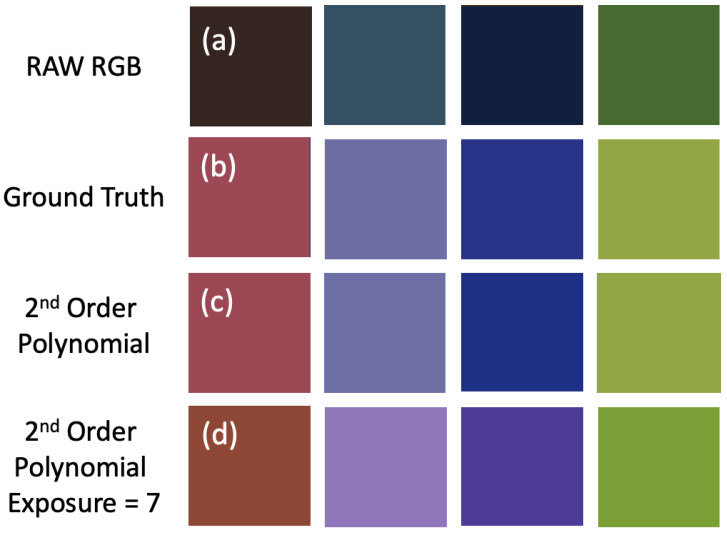
Top (**a**), four RAW RGB colour patches which are generated with Nikon D5100 camera sensitivities. (**b**), the true sRGB rendering of the patches. (**c**), the Nikon camera image corrected with a second-order polynomial regression. Bottom (**d**) shows the output of polynomial colour correction, calculated for exposure = 1, applied to the camera RGBs × 7 (exposure level = 7). All three images are scaled so the brightest value (across all three colour channels) is 1.

Let us now run a quick experiment to visually understand the problem of exposure in polynomial regression (we get similar results for the Neural Net). For the UEA dataset of spectral reflectance images [16], we sampled four reflectances. The RAW RGB responses of the Nikon D5100 are shown at the top of Figure 3a. Next, (b), the actual true sRGB image, rendered for a D65 whitepoint, is shown. In (c), we render these reflectances using the Nikon camera sensitivities and correct the four RGBs to the corresponding sRGB values using a second-order polynomial expansion. In detail, the second-order expansion has nine terms, ${\left[R^{2}\;G^{2}\;B^{2}\;RG\;RB\;GB\;R\;G\;B\right]}^{T}$, and the colour correction matrix is 9×3. In both the sRGB and fitted camera image, the maximum over all pixel values (across all three colour channels) is scaled to 1.

Now, we multiply the Nikon RGBs and the corresponding sRGB triplets by 7. As before, we calculate the second-order polynomial expansion of the RGBs and then apply the same colour correction matrix for the exposure = 1 condition. After colour correction, we again scale to make the exposure (across all three channels) equal to 1. The resulting colour patches are shown at the bottom of Figure 3. It is clear that the colours of the patches have changed significantly and that the colour correction is more accurate for the colours rendered under the same exposure conditions (panel (c) is more similar to (b) than (d) is to (b)).

In the second part of this paper, we try to solve this problem: we seek to make neural network colour correction exposure invariant. We investigate two approaches. First, we augment the training data used to define the neural network with data drawn from many different exposure levels. Second, we design a new network, which, by construction, is exposure invariant. Our new network has two components. The chromaticity component network attempts to map camera rgb chromaticity to colorimetric xyz chromaticity. In the second component, we linearly correct R, G and B to predict X + Y + Z (mean colorimetric brightness). Given this summed brightness and the target xyz chromaticity, we can calculate XYZ. By construction, the combination of the chromaticity-correcting network and the linear brightness predictor generates XYZs in an exposure-invariant manner. Experiments demonstrate that both of our exposure-invariant networks continue to deliver better colour correction than a 3 × 3 linear matrix.

In prior work in this area, color correction algorithms were assessed with respect to a single data set. For example, the 1995 reflectances (SFU set [17]) are widely used. Often *k* fold cross-validation is used, i.e., the reflectance set is split into *k* similar-sized folds. Then, each fold is used in turn as a test set and the other four are used for training the algorithms, and the performance of an algorithm is averaged over the *k* test sets. However, when a single set is used, often the statistics of all the folds are similar and so training on a subset of a given dataset can be almost as good as training on the whole set. Thus, an important contribution of this paper is to run a cross-validation experiment where the *folds* are different datasets (with known a priori statistics in common).

In Section 2, we summarise colour correction and introduce the regression and Neural Net (NN) algorithms. We show how NN methods can be made exposure invariant in Section 3 and we report on a comprehensive set of colour correction experiments in Section 4. The paper finishes with a short conclusion.

## 2. Background

Let $Q_{k}\left(\lambda \right)$ denote the k-th camera spectral response function and Q(λ) denote the vector of these functions as in Figure 1. The camera response to a spectral power distribution E(λ) illuminating the *j*-th reflectance $S_{j}\left(\lambda \right)$ is written as:

(2) $$\begin{array}{c} \rho =\int_{\omega }Q\left(\lambda \right)E\left(\lambda \right)S_{j}\left(\lambda \right)d\lambda \end{array}$$

where ω denotes the visible spectrum (400 to 700 nm) and ρ denotes the vector of RGB responses. Similarly, given the XYZ colour matching X(λ), the tristimulus response ***x*** is written as:

(3) $$\begin{array}{c} x=\int_{\omega }X\left(\lambda \right)E\left(\lambda \right)S_{j}\left(\lambda \right)d\lambda \end{array}$$

Suppose n×3 matrices *P* and *X* record (in rows) the camera responses and tristimuli of *n* surface reflectances, respectively. To find the 3×3 matrix *M*—that is, the best linear map—in Equation (1), we minimise:

(4) $$\begin{array}{c} \underset{M}{arg min}{∥PM-X∥}_{F} \end{array}$$

where ${∥.∥}_{F}$ denotes the Frobenius norm [18]. We can solve for *M* in closed form using the Moore–Penrose inverse [19]:

(5) $$\begin{array}{c} M={\left[P^{T}P\right]}^{-1}P^{T}X \end{array}$$

To extend the regression method, we define a basis function ${f_{e}}^{o}\left(\right)$ where the subscript *e* denotes the type of expansion—here *e = p* and *e = r*, respectively, denote polynomial and root-polynomial expansions—and the superscript *o* denotes the order of the expansion. As an example, if we are using the second-order root-polynomial expansion [5], then we write:

(6) $$\begin{array}{c} {f_{r}}^{2}\left(\rho \right)={\left[R\;G\;B\;\sqrt{RG}\;\sqrt{RB}\;\sqrt{GB}\right]}^{T} \end{array}$$

Again, we can use Equations (4) and (5) to solve for the regression matrix *M*, though *M* will be non-square (and depend on the number of terms in the expansion). For our second-order root-polynomial expansion, the columns of *P* will be the six terms in the root-polynomial expansion (*P* is a n × 6 matrix) and *M* will be 6 × 3. See, for example, [5] for details of higher-order expansions.

Optimising for the Frobenius norm in Equation (4) may be undesirable because the Euclidean differences in the XYZ colour space do not correspond to the perceived differences in colour. Instead, it is more desirable to optimise for the differences in perceptually uniform colour spaces such as CIELAB [2] or using colour difference formulas such as CIE Delta E 2000 [20]. Let us denote the magnitude of the difference vector between a mapped camera response vector and its corresponding ground truth CIELAB value as:

(7) $$\begin{array}{c} \Delta^{M,o,e}=∥C\left(M^{T}{f_{e}}^{o}\left(p\right),w\right)-C\left(x,w\right)∥ \end{array}$$

where *C*() maps input vectors according to the CIELAB function to corresponding Lab triplets and the superscripts *e* and *o* are as before. The parameter ***w*** denotes the XYZ tristimulus of a perfect white diffuser and is required to calculate CIELAB values. To find the best regression matrix, we seek to minimise:

(8) $$\begin{array}{c} \underset{M}{arg min}\sum_{i=1}^{n}\Delta_{i}^{M,o,e} \end{array}$$

Unfortunately, there is no closed-form solution to the optimisation described in Equation (1). Instead, a search-based strategy such as the Nelder–Mead simplex method [21] can be used to find *M* (though there is no guarantee that the global optimum result is found, [21] is a local minimiser).

In this paper, we will also be interested in minimising CIE Delta E 2000 [20]. Here, it is not possible to model colour difference as the Euclidean distance between triplets (which are non-linear transforms of XYZs and regressed RGBs). For the Delta E 2000 case, we write the error $\Delta_{2000}^{M,o,e}$ (subscript identifies the errors as CIE Delta E 2000) in Equation (9) and the minimisation to solve for is again given by Equation (8). As before, we minimise Delta E 2000 using the search-based algorithm. In Equation (9), we denote the function that calculates the CIE 2000 error as f(). We see this function takes three inputs: the regressed RGB ($M^{T}{f_{e}}^{o}\left(p\right)$), the corresponding XYZ (x) and the XYZ for the illuminant (w). For details of f(), the reader is referred to [20].

(9) $$\Delta_{2000}^{M,o,e}=f\left(M^{T}{f_{e}}^{o}\left(p\right),x,w\right)$$

**Figure 4:**
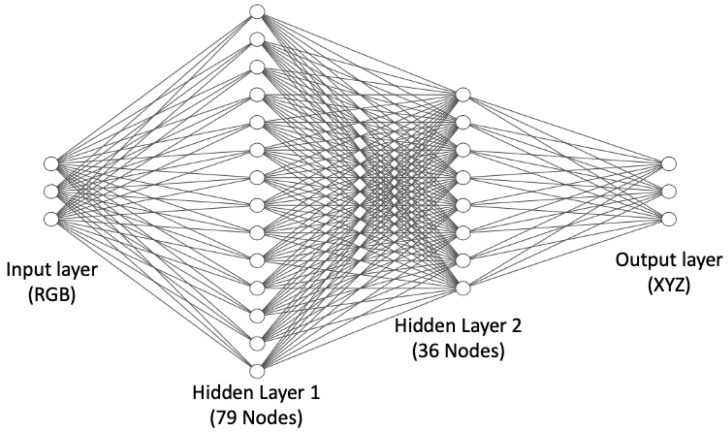
MacDonald and Mayer’s Neural Net [12]. Input and output layers consist of three nodes which are RGB and XYZ, respectively. In between, there are two hidden layers formed by 79 and 36 nodes.

As an alternative to regression methods, colour correction can also be implemented as an artificial neural network. MacDonald and Mayer’s [12] recently published neural network is illustrated in Figure 4 and is a leading method for neural network colour correction.

This Neural Net has 3189 ‘connections’, indicating the cost of colour correction is on the order of 3189 multiplications and additions (the number of operations applied as data flows from left to right). In comparison, the complexity of the second-order root-polynomial correction has three square root operations and (when the 6 × 3 correction matrix is applied) 18 multiplications and 15 additions, i.e., it is 2 orders of magnitude quicker to compute. In part, the practical utility or otherwise of the Neural Net approach will rest on the trade-off between how well it improves colour correction (say compared to the linear method) and its higher computational cost. The Neural Net is trained to minimise the Delta E 2000 errors.

## 3. Exposure-Invariant Neural Nets

Abstractly, we can think of a neural network as implementing a vector function f() such that:

(10) $$\begin{array}{c} f(\rho )\approx x \end{array}$$

where ρ and x denote the RAW RGB camera response vector and the XYZ tristimulus, respectively. When exposure changes—for example, if we double the quantity of light—then, physically, the RGB and XYZ responses also double in magnitude. We would like a colour correction function to be exposure invariant:

(11) $$\begin{array}{c} f(k\rho )\approx kx \end{array}$$

where *k* in Equation (11) is a positive scalar. This *homogeneity* property is actually rare in mathematical functions. It holds for linear transforms—f(ρ)=Mρ implies that f(kρ)=kMρ—and root-polynomials, but it is not true for polynomial expansions [5]. A Neural Net, in order to not collapse to a simple affine transformation, uses non-linear activation functions. These non-linearities, while an essential aspect of the network, make it difficult to attain homogeneity. This homogeneity is required if colour correction is to be invariant to a changing exposure level. The MacDonald and Mayer network is found not to be exposure invariant. In fact, this is entirely to be expected; there is no reason why NNs should exhibit the homogeneity property and every reason why they should not. Significantly, the variation in performance with exposure is a problem. In the experimental section, we show that colour correction performance drops markedly when there are large changes in exposure.

Now, let us consider alternative methods for enhancing the robustness of neural networks to exposure variations or achieving exact exposure invariance. In neural network research, if we observe poor performance for some input data, then the *trick* is to retrain the network where more of the problematic data are added to the training set. In neural network parlance, we *augment* the training data set. Here, we have the problem that a network trained for one light level delivers poor colour correction when the light levels change (e.g., when there is double the light illuminating a scene). So, to achieve better colour correction as exposure levels change, we will augment our colour correction training data—the corresponding RGBs and XYZs for a single exposure level—with corresponding RGBs and XYZs for several exposure levels. Our retrained MacDonald and Mayer Network using the exposure level augmented dataset is our first (more) exposure-invariant neural network solution to colour correction.

**Figure 5:**
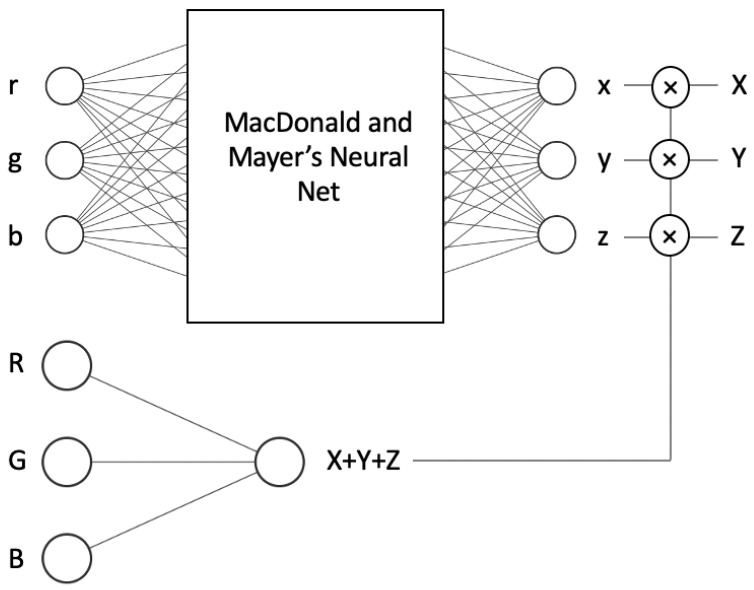
**NN-EI** with two networks, while the top one learns chromaticities, the bottom one learns the sum of XYZs. Multiplications of these give us the XYZs.

Perhaps a more elegant approach to solving the exposure problem is to redesign the network so it is, by construction, exactly exposure invariant. We show such an architecture in Figure 5. In the top network we learn—using Macdonald and Mayer’s **NN**—the mapping from input *r*, *g* and *b* chromaticity to *x*, *y* and *z* chromaticity. When the camera and tristimulus response are denoted as ${\left[R\;G\;B\right]}^{T}$ and ${\left[X\;Y\;Z\right]}^{T}$, then the corresponding chromaticities are defined as r=R/(R+G+B), g=G/(R+G+B) and b=B/(R+G+B); and x=X/(X+Y+Z), y=Y/(X+Y+Z) and z=Z/(X+Y+Z). In the ‘intensity’ network (bottom of Figure 5) we map *R*, *G* and *B* to predict X+Y+Z by using only a linear activation function. Multiplying the estimated ${\left[x\;y\;z\right]}^{T}$ by the estimated X+Y+Z returns an estimated ${\left[X\;Y\;Z\right]}^{T}$.

In other words, when we change the scalar *k*, the input of the first network remains unchanged, as it depends on the chromaticities. Consequently, the output also remains the same. The only elements that change are the input and output of the second (intensity) network, as it utilises the actual RGB input and calculates the sum of the actual XYZ. However, this change is linear since it involves a dot product; there are no activation functions or biases, only three multiplications. Thus, for the given input RGB × *k*, the multiplication of the output of the first network (xyz chromaticities) and the second network (intensity × *k*) yields (XYZ × *k*). As a result, the system is exposure invariant, meaning that chromaticities do not change at different illumination levels, and inputs and outputs scale linearly with the scalar *k*.

Given that the intensity network consists of only three connections with a linear activation function and no biases, the additional computational cost compared to the operation original network is quite low. It involves just one addition and six multiplication operations. Specifically, three multiplications and one addition are used for calculating the intensity, while the other three multiplications are performed to multiply the intensity with the x, y and x chromaticities in order to obtain the corresponding XYZ values.

Informally, let us step through an example to show that the network is exposure invariant. That is, we want to show that the respective RGBs ρ and kρ are mapped to the estimated XYZs *x* and kx. Let us consider the RGB vector [10, 50, 40]. To make the *r*, *g*, *b* chromaticities, we divide RGB values by the sum of RGB yielding the r, g, b chromaticities: [0.1, 0.5, 0.4]. Suppose our chromaticity network outputs [0.3, 0.4, 0.5] (the estimates of the *x*, *y*, *z* chromaticities) and the second network (the bottom one in Figure 5) returns 50 as the prediction of X + Y + Z. Now, we multiply output *x*, *y*, *z* chromaticities by 50, and we generate the XYZ output: [15, 20, 25].

Now, let us double the RGB values: [20, 100, 80]. Clearly, the chromaticities are unchanged ([0.1, 0.5, 0.4]). The output of the second network is a simple linear dot-product; thus, the output must be equal to 100 (as opposed to 50 before the exposure doubling). Finally, we multiply the estimated *x*, *y*, *z* chromaticities, [0.3, 0.4, 0.5], by 100 and the final output is [30, 40, 50] (which is exactly double as before). This simple example demonstrates that if the exposure changes by a scalar *k* then the output of the network also scales by *k* and so our new network is exposure invariant.

## 4. Experiments

### 4.1. Preparation of Datasets

In our experiments, we used four spectral datasets: the Simon Fraser University (SFU) reflectance set [17], the Ben-Gurion University Dataset (BGU) [22], the Columbia University Dataset (CAVE) [23], and the Foster et al. Dataset (FOSTER) [3]. The spectral sensitivities of the Nikon D5100 camera [24] and D65 viewing illuminant [25] are also used in all experiments. All input RGB values and their corresponding target XYZ values are calculated using numerical integration, without any gamma correction.

**Figure 6:**
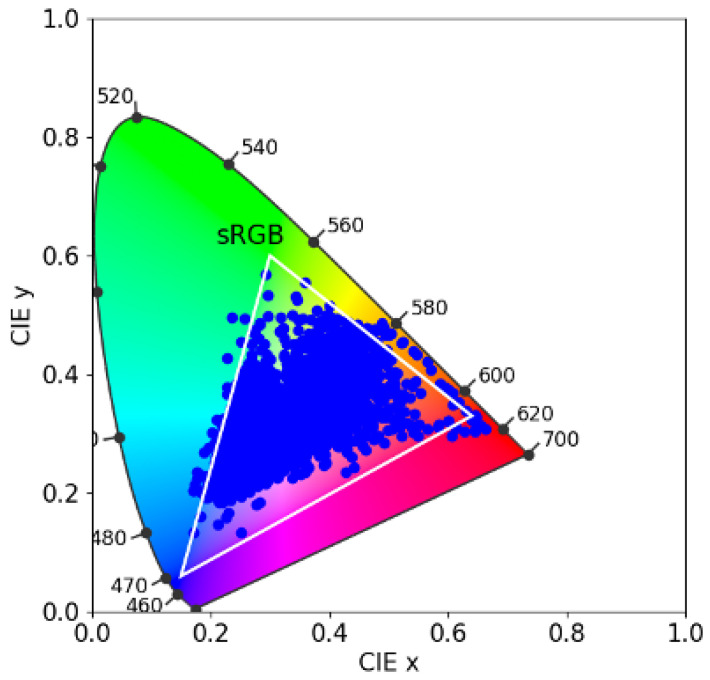
Gamut of the SFU dataset on the CIE 1931 chromaticity diagram. The white triangle shows the sRGB gamut.

The SFU reflectance set [17] comprises 1995 spectral surface reflectances, including the 24 Macbeth colour checker patches, 1269 Munsell chips, 120 Dupont paint chips, 170 natural objects, and 407 additional surfaces. In Figure 6, in the CIE 1931 chromaticity diagram, we plot the xy chromaticities of the SFU dataset. We also show the gamut of colours achievable using Rec 709 primaries (white triangle). It is evident that the SFU reflectance set comprises a wide range of colours.

The BGU Dataset comprises 201 multi-spectral outdoor images of various sizes. To make each image equally important (statistically), we resized them using bilinear resampling to 1000 × 1000 in size. In all our experiments, we used D65 as our viewing illuminant. However, the BGU images have radiance spectra and we would like the light component of each radiance spectrum to be D65. Thus, we set out to manually identify achromatic surfaces in each of the 201 scenes with the additional constraint that we judged the image to be predominantly lit by one light. We then used the corresponding spectrum (for the achromatic surface) to be the spectrum of the prevailing light. Dividing by the prevailing light and multiplying by D65 re-renders the scene for D65 illumination.

We were only confident that 57 of the 201 scenes met the two constraints of having a clearly identifiable achromatic surface and being lit by a single prevailing light. Thus, only 57 of the BGU images re-rendered to D65 were actually used. Finally, each image was scaled so that the maximum value in the image is 1.

The CAVE Dataset comprises 32 indoor reflectance images, segregated into five distinct categories: stuff, skin and hair, food and drinks, real and fake, and paints. Each image has dimensions of 512 × 512 pixels. We found it necessary to exclude one image, entitled “watercolors”, due to the presence of missing data. Consequently, we were left with a total of 8,126,464 pixels for analysis (31 × 512 × 512).

Lastly, we used the FOSTER hyperspectral image set [3] which consists of eight different images. Similar to the BGU Dataset, the various-sized images were resized to 1000 × 1000 and 8,000,000 pixels were obtained (8 × 1000 × 1000).

As the CAVE and FOSTER images contain only reflectance data, we multiply each spectral measurement in each image and per pixel by the D65 illuminant spectrum.

### 4.2. Algorithms

The following algorithms are investigated in this paper:

(i) **LS**: Least Squares Regression.
(ii) **LS-P**: denotes Least Squares Polynomial Regression. Here, we use the second-order expansion which maps each three-element vector to a ten-element vector.
(iii) **LS-RP**: Least Squares Root-Polynomial Regression. Again, a second-order expansion is used which for root-polynomials has six terms.
(iv) **LS-Opt**.
(v) **LS-P-Opt**.
(vi) **LS-RP-Opt**.
(vii) **NN**: MacDonald and Mayer’s Neural Net [12].
(viii) **NN-AUG**: The **NN** with an augmented training data with different exposure levels.
(ix) **NN-EI**: Here, we use two different neural networks. The first one learns to calculate chromaticities and the second one for the sum of XYZ.

**Opt** denotes optimisation, where we use the CIELAB or CIE Delta E 2000 loss values for training depending on the experiment (we make clear which is used in which experiment) with the Nelder–Mead simplex method [21] (see Equations (7) and (8)) to solve for the linear (iv), polynomial (v) and root-polynomial regressions (vi). The regressions (i) through (iii)—minimising error in the XYZ tristimuli space—are found in closed form using the Moore–Penrose inverse (Equations (5) and (6)).

As suggested in MacDonald and Meyer’s original paper all the colour corrections, NNs are trained to minimise the CIE Delta E 2000 error.

Apart from the cross-dataset experiments, our colour correction algorithms are tested on the SFU dataset using a five-fold cross-validation methodology for the fixed and different exposures experiments. Here, the reflectance dataset is split into five equal-sized folds (399 reflectances per fold). Each algorithm is trained using four of the folds and then tested on the remaining fold to generate error statistics. The process is repeated five times (so every fold is the test set exactly once). According to this methodology, the reported error statistics are averages over the five experiments.

### 4.3. Details about How the Neural Net Was Trained

For **NN**, we used MacDonald and Mayer’s [12] neural network, which has RGB values as input and XYZ as target, the 3 × 79 × 36 × 3 fully connected MLP architecture with two hidden layers as shown in Figure 4. As in the original paper, we used the Adam optimiser with a learning rate of 0.001 to train the network to minimise CIE Delta E 2000 [20]. We had to raise the number of epochs from 65 (used in the original study) to 500 for the neural network to develop a successful mapping because we were working on relatively small datasets. We also used mini-batch gradient descent with a batch size of 8. Our model used 20% of the training data for the validation set and used the early stopping method, which means that the training ends automatically if there is no improvement in validation loss after a specified number of epochs (which in our model is 100) with a call-back function. We chose the best model based on the validation loss. The **NN-AUG** and **NN-EI** were trained using the same methodology.

### 4.4. Details about Exposure Experiments

The colour correction performance for all algorithms was first calculated for a fixed reference exposure (exposure = 1) level. Then, we tested our models under different exposure levels to understand their performance when the exposure level changed. We use exposure values of 0.2, 0.5, 1, 2 and 5 (e.g., 0.2 and 5, respectively, meaning the amount of light was 1/5 and 5 times the reference condition of exposure 1).

In **NN-AUG**, in order to achieve successful results at different exposure levels, we augmented the training data with different exposure factors, which are 0.1, 0.2, 0.5, 2, 5 and 10 times the original samples. Then, we tested the models with the test samples with an original exposure level.

The **NN-EI** is, by construction, exposure invariant; thus, it was only trained using the data for the reference exposure level.

### 4.5. Details about Cross-Dataset Experiments

In the cross-dataset section, we trained our algorithms with a single dataset and individually tested them with the remaining datasets. We repeated this process four times (i.e., every dataset was used for training once and for testing three times). Additionally, for the NN method, we allocated 20% of the training data as a validation set. The whole training set was used for the regression colour correction methods.

Even our relatively small image sets comprise millions of pixels. Training with millions of pixels is computationally expensive, especially for the NN and search-based regression methods. To mitigate training complexity, we reduced the multi-spectral images of the BGU, CAVE, and FOSTER datasets to thumbnails of size 40 × 40 × 31 (using nearest neighbour downsampling). Additionally, for all our datasets (including SFU), we sampled the set of images and included an observed spectrum if and only if it was at least 5 degrees apart from any spectrum already in the set (we built the set incrementally by adding a new spectrum if it was 5 degrees away from all previously selected members).

Thumbnail creation and angular threshold filtering resulted in, respectively, 523, 354, 4188 and 4331 spectra for the SFU, BGU, CAVE and FOSTER datasets. All our colour correction algorithms were trained for these small sets of spectra. However, testing was conducted on the full-sized spectral data, which were 1995 samples for SFU, 57,000,000 for BGU (57 × 1000 × 1000), 8,126,464 for CAVE (31 × 512 × 512) and 8,000,000 for FOSTER (8 × 1000 × 1000).

## 5. Results

### 5.1. SFU Results for the Fixed Illumination

We report the CIELAB and CIE Delta E 2000 error results for our seven algorithms for the SFU dataset in Table 1 and Table 2, respectively. As discussed earlier, we ran a five-fold cross-validation experiment. This means that there are five sets of colour correction—four folds used for training and the other for testing. We calculate the Mean, Max, Median and 95% (percentile) errors across five folds. The figures in Table 1 and Table 2 report these error statistics averaged over the five sets. For the ‘-Opt’ algorithms, we minimised the mean CIELAB Delta E error and then in Table 1, we assessed the correction performance also using CIELAB. Table 2 reports the same result, where Delta E 2000 was used (but again the algorithms were trained to minimise the CIELAB error.

We see that, although the neural network algorithm returns significantly better results compared with the standard Least Squares Regression model, it delivers poorer colour correction compared with the other regression methods. The polynomial and root-polynomial deliver low errors, and both can be improved using the differences in the CIELAB space as a loss function. The -Opt variants of the regression algorithms have significantly lower maximum errors. The reader might be interested to know that if we train the neural network with the CIELAB loss function or use CIE Delta E 2000 on the classical methods, we found that these do not change the rank ordering of the results.

**Figure 7:**
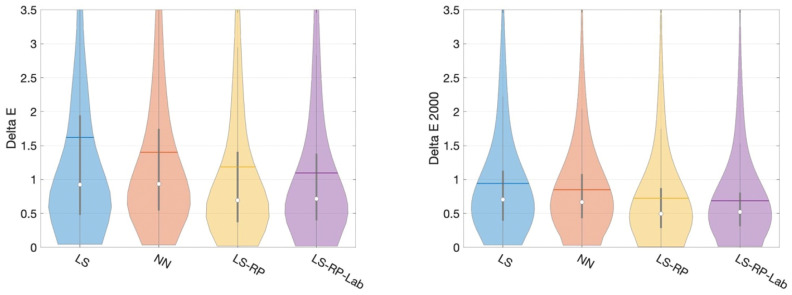
A comparison of error distribution of four main methods by CIE Delta E and CIE Delta E 2000. Inside each ‘violin’, the white dot, the horizontal line, the vertical bar and the black line indicate, respectively, the median, mean, interquartile range and 1.5 interquartile range.

In Figure 7, we plot the CIELAB and CIE Delta E 2000 error distributions as violin plots for four of our algorithms. The tails of the violin plots are long because of the large maximum values (outliers). The width of the violin captures the probability density of the error distribution [26]. Again, the distributions show that the root-polynomial regression with the CIELAB loss shows the best performance.

We performed the sign test [27] to establish if there is a statistically significant difference between the NN and LS-RP-Opt results. Both the p-values for CIELAB and CIE Delta E 2000 are less than 0.0001 in the 99% confidence level. The difference in algorithm performance is statistically significant.

### 5.2. Results for Different Exposure Values with Exposure Invariant Neural Nets

In Table 3, we report the performance results of the six methods at different illumination levels. As a reminder, in this experiment, the algorithms were trained with the original light level and tested with different intensity levels. The linear and root-polynomial regression methods are unaffected by exposure (they have the same performance across exposure changes). Equally, it is evident that the original MacDonald and Meyer **NN** performs poorly when the exposure changes.

Although the **NN-AUG** method exhibits a fair degree of exposure invariance, its performance still degrades slightly as the change in light levels is more extreme (compared to the reference condition). The **NN-EI** that was designed to be exactly exposure invariant delivers better results than the **NN-AUG** method, especially for the conditions where the exposure level is smaller than 0.5 or bigger than 5.

### 5.3. Results for Cross-Dataset Experiments

Table 4, Table 5, Table 6 and Table 7 present the test results of the seven algorithms trained on the SFU, BGU, CAVE, and FOSTER datasets, respectively (and then tested on the combination of the three remaining datasets). The regression models labelled as ‘Opt’ in this section were optimised using the CIE Delta E 2000 loss function, similar to the NN. All results displayed in the tables represent the average CIE Delta E 2000 errors. It is worth noting that to avoid overwhelming the article with results, we did not include tables containing CIELAB results. We used CIELAB to train the algorithms previously and here we use CIE 2000. Irrespective of the colour difference metric used to train or test the methods, the ranking of the algorithms stays the same.

Regarding Table 4, we observe the cross-dataset test results of models trained with SFU. All models demonstrate their best performance on the FOSTER dataset, followed by the CAVE dataset, and finally on the BGU dataset. However, regardless of the test data, LS-P consistently remains the top-performing model, while NN exhibits the poorest performance. The change in the ranking of polynomial and root-polynomial algorithms is not entirely unexpected. Here, there is a greater difference in the spectra in training and testing datasets. The previous experiments might be interpreted as that the RP method works well when training and testing datasets share similar spectral statistics.

In Table 5, we trained using the BGU dataset and tested on the other datasets. Here, both polynomial models and the NN performed poorly. The best method overall is the Linear 3 × 3 matrix (LS).

Table 6 reports the results of models which are trained on the CAVE Dataset and tested on other datasets. Here, we observe that models trained on the CAVE Dataset yield better results compared to the other tables. This might indicate that CAVE best represents the data found in the other test sets. Here, the root-polynomial method performs best.

In Table 7, we examine the training results using the FOSTER dataset. The best models for SFU, BGU and CAVE were LS, LS-Opt and LS-P-Opt, respectively.

Table 4, Table 5, Table 6 and Table 7 reveal several interesting findings. First, when the statistics of training and testing data are different, there is not a clear ranking in the algorithms in general. Second, even though the testing methodology does not contain a change in exposure, we find that the NN method delivers poor performance, most likely due to overfitting. The algorithm that generalises to other datasets is always one of the classical regression methods and never the NN method.

## 6. Conclusions

Recently, it has been proposed that neural networks can be used to solve the colour correction problem [12]. Indeed, in line with previous work, we found the **NN** approach delivered a modest performance increment compared to the (almost) universally used linear correction method (at least where training and testing data contain similar spectral data) [13]. However, we also found that the **NN** approach was not exposure invariant. Specifically, a network trained for one light could actually deliver poor colour correction as the exposure changed (there was more or less light in the scene).

However, we showed that NNs could be made robust to changes in exposure through data augmentation by training the NNs with data drawn from many different light levels. In a second approach, we redesigned the neural network architecture so that, by construction, it was exactly exposure invariant. Experiments demonstrated that both exposure-invariant networks continued to outperform linear colour correction. However, a classical method—the simple exposure-invariant root-polynomial regression method—worked best overall (outperforming the **NN** by about 25%).

Finally, we carried out cross-dataset experiments to test the performance of our algorithms. This is a stringent test as the spectral statistics of the datasets used are quite different from one another. Our results showed that there is not a clear ranking in the performance of regression-based methods for the cross-dataset condition. However, as for the exposure change test, we found that the NN method performed worse overall.

The general conclusion of this paper is that—at least for now—classical colour correction regression methods outperform the tested NN algorithm.

## Figures and Tables

**Table 1:** CIE LAB Delta E error statistics.

Methods	Mean	Max	Med	95%
LS	1.62	15.47	0.93	5.32
LS-P	1.29	11.00	0.78	4.01
LS-RP	1.19	13.97	0.70	3.62
LS-Opt	1.48	10.62	0.9	4.63
LS-P-Opt	1.17	8.47	0.77	3.62
LS-RP-Opt	1.10	7.36	0.72	3.39
NN	1.40	12.26	0.93	4.06

**Table 2:** CIE Delta E 2000 error statistics.

Methods	Mean	Max	Med	95%
LS	0.94	7.71	0.70	2.62
LS-P	0.79	4.63	0.59	2.18
LS-RP	0.72	7.13	0.49	2.14
LS-Opt	0.91	5.52	0.67	2.45
LS-P-Opt	0.75	4.25	0.54	2.08
LS-RP-Opt	0.69	3.81	0.52	1.96
NN	0.85	4.18	0.66	2.15

**Table 3:** CIE LAB Delta E error statistics at different exposure levels.

Methods	0.2	0.5	1	2	5
LS	1.62	1.62	1.62	1.62	1.62
LS-RP	1.19	1.19	1.19	1.19	1.19
LS-RP-Opt	1.10	1.10	1.10	1.10	1.10
NN	2.60	1.57	1.40	1.92	3.77
NN-AUG	2.25	1.50	1.30	1.25	1.38
NN-EI	1.53	1.53	1.53	1.53	1.53

**Table 4:** Mean Delta E 2000 error statistics (SFU used as training set).

Methods	BGU	CAVE	FOSTER
LS	3.49	4.60	2.49
LS-P	3.12	4.28	2.03
LS-RP	3.52	4.56	2.36
LS-Opt	3.49	4.69	2.32
LS-P-Opt	3.31	4.52	2.04
LS-RP-Opt	3.46	4.47	2.28
NN	3.93	4.86	2.51

**Table 5:** Mean Delta E 2000 error statistics (BGU used as training set).

Methods	SFU	CAVE	FOSTER
LS	3.31	4.45	2.95
LS-P	10.80	6.59	5.64
LS-RP	5.27	5.88	2.81
LS-Opt	3.81	4.30	3.37
LS-P-Opt	10.85	6.61	5.63
LS-RP-Opt	5.51	5.58	3.16
NN	17.10	18.75	21.98

**Table 6:** Mean Delta E 2000 error statistics (CAVE used as training set).

Methods	SFU	BGU	FOSTER
LS	2.15	1.88	2.54
LS-P	2.43	1.47	2.57
LS-RP	1.91	1.58	2.48
LS-Opt	4.11	3.69	2.98
LS-P-Opt	3.26	1.74	3.21
LS-RP-Opt	3.75	3.39	2.83
NN	2.61	2.02	3.57

**Table 7:** Mean Delta E 2000 error statistics (FOSTER used as training set).

Methods	SFU	BGU	CAVE
LS	2.38	2.44	4.77
LS-P	2.58	2.47	4.20
LS-RP	3.12	2.40	4.38
LS-Opt	2.49	1.96	4.30
LS-P-Opt	2.82	3.01	3.96
LS-RP-Opt	2.91	1.98	4.18
NN	5.62	5.52	4.71

## Data Availability

Four publicly available datasets were used in this study. First, SFU’s surface reflectance dataset: https://www2.cs.sfu.ca/~colour/data/colour_constancy_synthetic_test_data/ (Accessed date: 4 October 2023). Second, BGU’s multispectral dataset which can be accessed via: https://icvl.cs.bgu.ac.il/hyperspectral/ (Accessed date: 4 October 2023). Third, the CAVE dataset which can be accessed via: https://www.cs.columbia.edu/CAVE/databases/multispectral/ (Accessed date: 4 October 2023). Fourth, the FOSTER dataset which can be accessed via: https://personalpages.manchester.ac.uk/staff/d.h.foster/Hyperspectral_images_of_natural_scenes_04.html (Accessed date: 4 October 2023).
